# Decomposing the variance in early maladaptive schemas: the major role of one general factor, the minor role of domains, and their differential relations to facial emotion recognition

**DOI:** 10.3389/fpsyg.2024.1342480

**Published:** 2024-05-15

**Authors:** Sajedeh Tabesh, Ali Zia-Tohidi, Manijeh Firoozi, Hojjatollah Farahani

**Affiliations:** ^1^ Department of Psychology, Faculty of Psychology and Educational Sciences, University of Tehran, Tehran, Iran; ^2^ Department of Psychology, Faculty of Humanities, Tarbiat Modares University, Tehran, Iran

**Keywords:** schema therapy, facial emotion recognition, factor analysis, psychometrics, early maladaptive schemas

## Abstract

**Introduction:**

Despite the growing interest in the early maladaptive schemas, the progress in understanding their impacts is decelerated by a lack of clear understanding of their structure. Different composite scores are calculated without a solid ground or a clarified meaning. Here we explain that the schema variance can be theoretically decomposed into three components: schema-specific, domain-specific due to the unmet core needs, and the common variance we call general susceptibility; each can differentially correlate with other substantive variables. Using this framework, we empirically examine the structure of schemas and their relationships to facial emotion recognition, a crucial ability that can widely affect our social interactions.

**Methods:**

A sample of adults completed an emotion recognition task and the Young Schema Questionnaire. Using different factor models, the specific and shared variance across schemas was analyzed. Then, the relation of these variance components to facial emotion recognition was explored.

**Results:**

A general factor explained 27%, 40%, and 64% of the total variance in items, schemas, and domains, respectively. Partialling out the common variance, there was little domain-specific variance remained. Regarding facial emotion recognition, they were not correlated with specific schemas; however, the general susceptibility factor was correlated with anger recognition.

**Discussion:**

The variance decomposition approach to schemas, which uses the bifactor model, may offer a clearer way to explore the impacts of schemas. While domain scores are widely used, their reliability, validity, and meaning are questionable. The generic factor, which is consistently extractable from empirical data, requires further attention.

## 1 Introduction

Our early experiences undisputedly influence our later life perception and behavior. As infants, our brain is at its greatest flexibility to learn how the environment operates. It detects systematic patterns within seemingly random noisy events (Strack and Deutsch, 2004). Based on these patterns, we shape sets of schemas that guide us for better adjustment to our environment. However, as the brain’s flexibility declines with age, these schemas become more fixed and rigid. Once adaptive, some become maladaptive later in life and harmful to our wellbeing. This is how early maladaptive schemas (EMSs) are developed; they are developed in response to the frustration of core emotional needs and then persist throughout our lives (Young et al., 2003, p. 10; Bach et al., 2018).

### 1.1 EMSs, social life, and facial emotion recognition

Given the basics of the theory, the core emotional needs and the EMSs are both about our interpersonal life. While most studies have focused on their influence on more complicated constructs such as depression and interpersonal problems, recently some researchers have proposed that schemas may affect our interpersonal functions at a more basic level: our ability to recognize emotions from facial cues (Csukly et al., 2011; Panić et al., 2022). For instance, a person with an Abandonment schema may be more sensitive to facial cues of disgust; another with a Vulnerability schema may be more sensitive to anger; a third person with a Self-Sacrifice schema may be oversensitive to sadness; and one with an Insufficient Self-Control schema who has a high tendency to avoid discomfort (Young et al., 2003, p. 16) may automatically avoid facial cues of sadness or anger. Though these relations make logical sense, they are speculative and hence need empirical validation. If such hypotheses are correct, the maladaptively filtrated nonverbal cues may adversely affect our social life, given the vital role of nonverbal communication (Wegrzyn et al., 2017).

The influence of early experiences on facial emotion recognition fits even biologically-based theories such as Ekman’s theory (Ekman, 1992). While considering emotion recognition as biologically determined, Ekman argues that humans have an *open affect program*. That is, their program is open to inputs from their experience; once these new inputs are integrated into the program, it runs automatically as it was biologically determined (Ekman and Cordaro, 2011).

### 1.2 Schemas, schema domains, and the general susceptibility factor

Young Schema Questionnaire (YSQ) is the primary tool for assessing the EMSs. Given its theoretical framework and the empirical evidence from factor analytic studies (e.g., Bach et al., 2018), a two-level structure is suggested for the YSQ.

The first-order structure corresponds to the EMSs; each latent factor is assumed to indicate the severity of a schema. As there are many schemas (18 in YSQ-3), it is difficult to study how they relate to other substantive variables. Therefore, many researchers prefer to reduce the schemas to a smaller number of variables, i.e., the second-order factors.

The second-order structure, called schema domains, is theoretically based on categorizing schemas according to unmet needs that give rise to the EMSs. Young et al. (2003, p. 10) tentatively proposed that there are five core emotional needs: secure attachment, autonomy and competence, freedom to express needs and emotion, spontaneity and play, and realistic limits and self-control. Accordingly, the frustration of one need leads to the formation of one or more maladaptive schemas within its corresponding domain. They did not, however, clarify how the frustration of one of these five core needs can lead to not only a specific one but to different schemas within a domain. We can think of at least two hypotheses. The first is that these five needs are further subdivided into facets, and the frustration of each facet of these needs can lead to a specific schema. The second hypothesis is that these needs are unidimensional, but other factors such as temperament determine which EMS to form.

Taking a more in-depth look into the theoretical perspectives, clinical experience, and empirical evidence on core emotional needs, Lockwood and Perris (2012) suggested that for every single schema, there is an unmet need. They further suggested a hierarchical structure for these needs, which they classified into four broad domains. As the schemas stem from the needs, their structure is assumed to parallel the structure of needs. Coauthored by Young, Bach et al. (2018) confirmed this four-domain structure of EMSs on a large sample of clinical and non-clinical participants. Thimm (2022) also provided meta-analytic evidence that this structure is the best fit. Further support came along when a fairly comparable structure was found in early adaptive schemas as well (Louis et al., 2020); that is if the needs are responsible for both maladaptive and adaptive schemas, the same structure is expected to be found in both.

While researchers are attracted to the use of second-order structure (as it reduces the large number of schemas to a more manageable number), there are at least two major issues that need to be addressed. First, the meaning of the higher-order constructs is not well clarified yet. The domain-level scores reflect what is shared between groups of schemas. If we assume their correspondence with the needs hierarchical structure, we then must clarify what the need domains really mean. If we assume the original five core needs, with different schemas rising from a combination of unmet needs and other factors, then the shared component may have a very different meaning. The second problem is, as we will show here, that some domains substantially overlap, to the extent that they may not be easily differentiable; therefore they may not be useful constructs.

Besides the first–and second-order structures, another level of structure also makes sense, as there seems to be a common susceptibility factor across all schemas. Extensive evidence from gene–environment interaction (GxE) indicates that individuals are not similar in how they are affected by the environment. Some are very sensitive to both risky and supporting environments, likely due to higher levels of neuroplasticity; some are less so (Belsky et al., 2009; Boyce, 2016; Assary et al., 2018). On the other hand, neglective environment tends to neglect different aspects of the child’s needs. Hence, a common variability is generated, where sensitive individuals with poor environments develop different maladaptive schemas, while less sensitive individuals do not.

For instance, the development of different schemas is likely affected by similar temperamental and environmental factors such as neuroticism, and poor parenting modes (Lockwood and Perris, 2012). For instance, someone who this is further evident from the positive correlation among all schemas (for a meta-analysis see Thimm, 2022).

Given what we elaborated, and taking a statistical perspective, the reliable variance in schemas can be decomposed into three components: (1) the variance due to the general susceptibility that is shared among all schemas and/or schema domains; (2) the variance due to how the interaction between general susceptibility and environment leads to the frustration of some needs but not others; and (3) the variance in the specific schemas developed in response to the frustration of needs. These different variance components may differentially correlate with other variables of interest; therefore, ignoring the issue can lead to the misinterpretation of empirical findings, hindering the advancement of our understanding of the underlying mechanisms.

### 1.3 Current study

In this study, we first explore the structure of schema, with a greater focus on schema domains and the general susceptibility factor. While such a generic factor has been suggested and investigated before (Kriston et al., 2012; Oettingen et al., 2018; Yan et al., 2018), the possibility that it may constitute the major proportion of variance in domain scores has not been explored. This is important for research, given the widespread interest in using domain scores. As a second objective, we used the variance decomposition approach to explore how different variance components of schemas relate to facial emotion recognition, a fairly under investigated topic (Csukly et al., 2011; Panić et al., 2022). We suggest that such a framework may facilitate a more in-depth and less biased understanding of how schemas are related to other constructs of interest, especially with regard to the general susceptibility factor which seemingly has not been explored.

## 2 Methods

### 2.1 Participants

A total of 259 participants were recruited from social media; snowball sampling was implemented when possible. All participants were Iranian adults (> 18) with no severe mental disorders. A data cleaning procedure, using Mahalanobis distance (Desimone et al., 2015), revealed 26 potentially random responders (*p*s < 0.001). Removing these cases yielded a sample of 233. Of the final sample, the majority were female (88%), unmarried (68%), and with academic degrees of bachelor’s or higher (70%). The age distribution was positively skewed, with a median of 22 and a range of 18–71; 73% were between 18 and 30 and 27% were older.

### 2.2 Power analysis

To ensure sufficient power, we conducted a series of power analyses, using the semPower package (Moshagen and Erdfelder, 2016). These analyses show us the present statistical power to reject the models due to global model misfit, based on a commonly used approximate fit index, root mean square of error approximation (RMSEA). For the original first-order structure, our sample of 233 had excellent power (> 0.99) to detect a misfit of RMSEA = 0.05 (*α* = 0.05, *df* = 2,595). For the original second-order structure, our sample of 226 (excluding schema-level outliers) had a power of 0.9 to detect such a misfit (*α* = 0.05, *df* = 80). Concerning hypotheses about relations, we had 0.8 power to detect a significant (*α* = 0.05) correlation of |r| = 0.185 (*r^2^* = 0.034; this power analysis was conducted in G^*^Power. Given the complexity of power analysis in the context of SEM, we used the traditional power analysis as an approximation).

### 2.3 Instruments

#### 2.3.1 Young Schema Questionnaire

The 75-item Young Schema Questionnaire (YSQ-75) is designed to assess 15 EMSs (Young, 1998). Its Persian version has been validated in Iran and has shown acceptable reliability (e.g., Khosravani et al., 2020). In this study, the reliability was good to excellent, with McDonald’s *ω* coefficients ranging from 0.79 to 0.92 (Supplementary Table S1).

#### 2.3.2 Facial emotion recognition task

**Figure 1:**
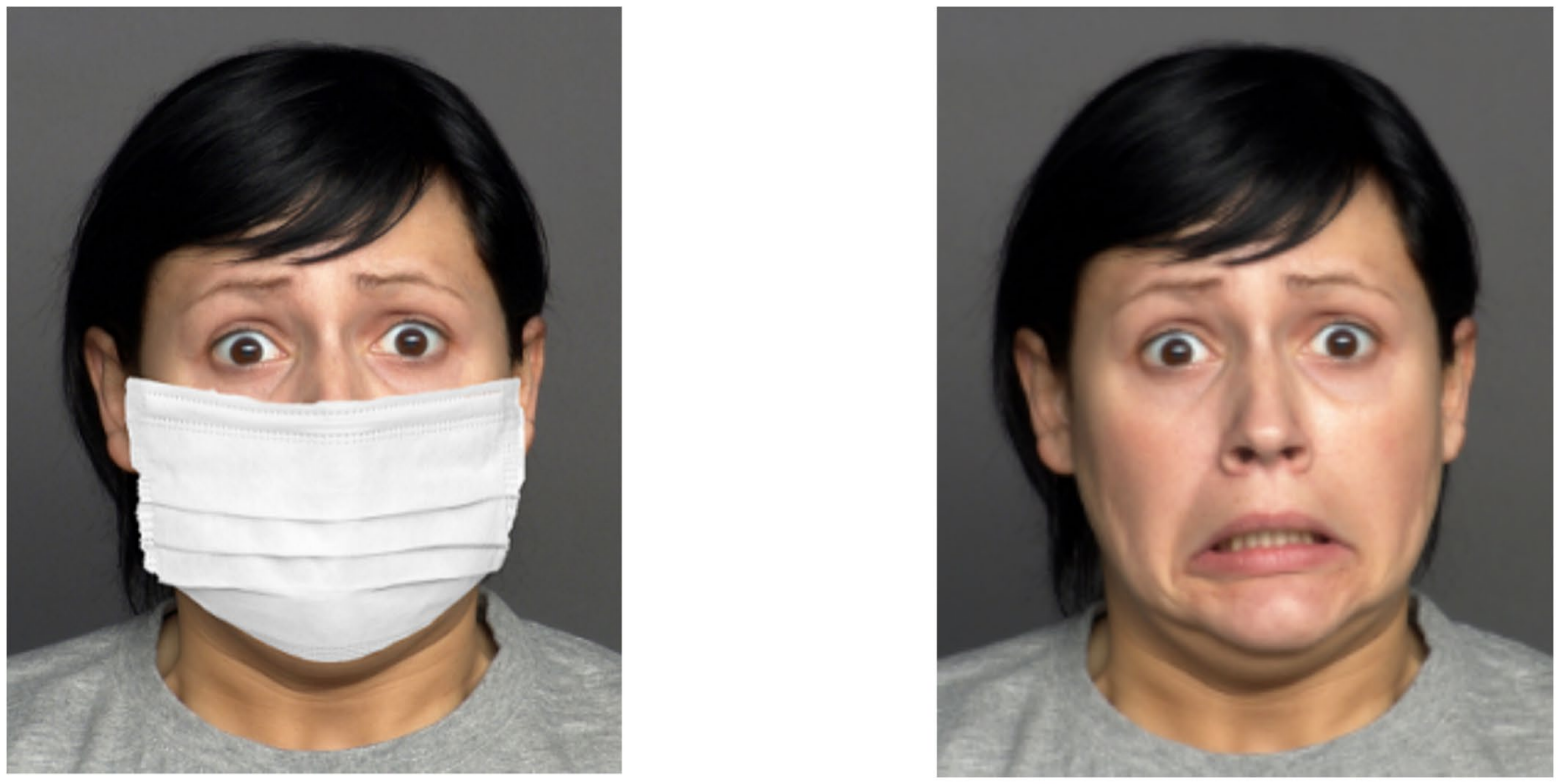
Samples of pictures of emotional faces with and without the mask (fear emotion in female face), the non-masked face image stems from the MPI database (Ebner et al., 2010).

Each participant was presented with 24 facial emotion recognition conditions in the form of static pictures: six emotional states (fear, disgust, anger, happiness, sadness, and neutral) × 2 masking conditions (masked vs. non-masked) × gender (male vs. female). The pictures were selected from the MPI database (Ebner et al., 2010; Figure 1). For masked conditions, the mask was added to the pictures to cover the nose and mouth. Participants had to select the correct response among the six possible choices. The two-way mixed-effect intra-class correlation (ICC; Koo and Li, 2016) for the total score (aggregated across 24 conditions) was acceptable, given the nature of the task, ICC = 0.61. However, the ICC for scores on separate emotions (each aggregated across four conditions) was not good, therefore, the results regarding separate emotions must be interpreted with more caution (disgusted: 0.31; angry: 0.34; happy: 0.55; sad: 0.43; fearful: 0.15; and neutral: 0.27).

### 2.4 Procedure

The research was conducted in 2022. The data was collected via Google Forms; the link was distributed through social media (e.g., WhatsApp, and Instagram). Participants completed demographics, then YSQ–75, followed by the facial emotion recognition task. They provided consent by clicking a checkbox with a clear description. If they were willing so, they received their results on YSQ via email.

### 2.5 Statistical analysis

All analyses were conducted in R. A data cleaning procedure was conducted, using Mahalanobis distance, to detect random responders; a probability of *p* < 0.001 was considered to indicate random responding (DeSimone et al., 2015). We used lavaan (Rosseel, 2012) to perform confirmatory factor analysis (CFA) and structural equation modeling (SEM). Due to moderate violation of parametric assumptions (normality and homoskedasticity), the robust maximum likelihood (MLR) was used for estimation, along with sandwich-type standard error and robust fit indices [χ^2^, comparative fit index (CFI), root mean square of error approximation (RMSEA) with its 90% confidence intervals, and standardized root mean square residual (SRMR)]. For higher-order factor analysis, we included schema scores as indicators to be able to assess the fit exclusively for higher-order structure.

## 3 Results

### 3.1 Data screening

We explore the distributional characteristics of the data to ensure the statistical assumptions. The item-level data on the Yaung’s questionnaire showed skewness indices ranging from −0.1.24 to 3.47; however, only two items showed skewness higher than 3. They also showed kurtosis indices (ranging from −1.37 to 13.52). Expectedly, the item-level data further showed heteroskedastic bivariate distributions. The schema-level data also showed mild to moderate violations of normality (with skewness ranging from 0.01 to 2.37 and kurtosis from −0.56 to 8.9) and homoskedasticity, though these violations were less severe. Due to these data characteristics, to ensure valid results we used the method of Robust Maximum Likelihood with a heteroskedasticity consisted standard error along with robust fit indices.

### 3.2 The structure of schemas

#### 3.2.1 The first-order structure

To evaluate the first-order structure, we first fitted a one-factor model, on which all items were loaded. The fit was poor (Table 1); even so, all loadings were positive, 80% of loadings were 0.4 or higher, the median loading was 0.57, and the factor accounted for 28.5% of the total variance in all items. We next fitted the original 15-factor model, which showed an acceptable fit, RMSEA_(robust)_ = 0.057, 90% CI [0.054, 0.06], SRMR_(robust)_ = 0.077. Although the CFI_(robust)_ was 0.81, in our model this was not problematic, as the null model RMSEA was low (RMSEA_(null)_ = 0.129) and, in such situations, incremental fit indices do not necessarily indicate poor fit (Kenny, 2020). With one exception, all items showed acceptable loadings on their corresponding factors (all βs > 0.46).

**Table 1:** Model fit for first–and second-order structures of Young Schema Questionnaire–Short Form.

Model	χ^2^	df	CFI	SRMR	RMSEA [90% CI]
First-order structure					
1 factor	7673.2	2,700	0.494	0.098	0.092 [0.09, 0.094]
15 uncorrelated factors	6274.9	2,700	0.641	0.273	0.078 [0.075, 0.08]
15 correlated factors	4464.9	2,595	0.813	0.077	0.057 [0.054, 0.06]
Bifactor: 1 general and 15 uncorrelated factors	4592.9	2,625	0.804	0.078	0.058 [0.055, 0.061]
Second-order structure					
1 factor	451	90	0.757	0.092	0.141 [0.128, 0.154]
5 factors^a^	348.2	80	0.824	0.082	0.127 [0.114, 0.141]
4 factors—a	331.6	84	0.838	0.086	0.119 [0.106, 0.133]
4 factors—b	296.6	83	0.857	0.078	0.113 [0.099, 0.127]
4 factors—orthogonal bifactor—a^b^	234.4	74	0.894	0.072	0.103 [0.088, 0.118]
4 factors—orthogonal bifactor—b^b^	201.7	73	0.915	0.052	0.093 [0.078, 0.108]
4 factors and one third-order factor	358.3	86	0.82	0.082	0.124 [0.111, 0.138]

First-order structure models *N* = 233; second-order structure models *N* = 226. Robust maximum likelihood is used for all models; all fit indices are robust. CFI, Comparative fit index; SRMR, Standardized root mean square residual; RMSEA, Root mean square error of approximation. ^a^This model had two problems, a non-positive definite covariance among latent factors and a negative variance in approval seeking. Relaxing two residuals to freely covary, resolved the problem. The fit was better, χ^2^(78) = 268.3, *p* < 0.001, CFI (robust) = 0.875, SRMR (robust) = 0.072, RMSEA_(robust)_ = 0.109 [0.95, 0.123]. ^b^For this model to converge, we had to allow Disconnection and Rejection to freely covary with Impaired Autonomy.

We next fitted a bifactor model with a general factor loaded by all items and 15 uncorrelated specific factors. The global fit was fairly similar to the 15-factor model (Table 1). Notably, the common factor accounted for 27% of the total variance in all items, and the 15 specific factors accounted for 27%. The Sattora-Bentler’s test and AIC, favored the 15 correlated factors model over the bifactor model, Δχ^2^(30) = 128.5, *p* < 0.001, ΔAIC = 70; however, ΔBIC = −29 favored the bifactor model. In terms of reliability, the general factor had good reliability, *ω* = 0.83, but only three specific factors had *ω* coefficients higher than 0.6 (see Supplementary Table S1).

#### 3.2.2 The higher-order structure

Before analyzing the data for second-order structure, seven schema-level multivariate outliers were removed (Mahalanobis distance values with *p* < 0.001), yielding a sample of 226. We first fitted a single-factor second-order structure. While the fit was poor (Table 1), the factor loadings were interestingly high; all loadings were above 0.3 and the single factor accounted for 40% of the total variance in all schemas.

**Figure 2:**
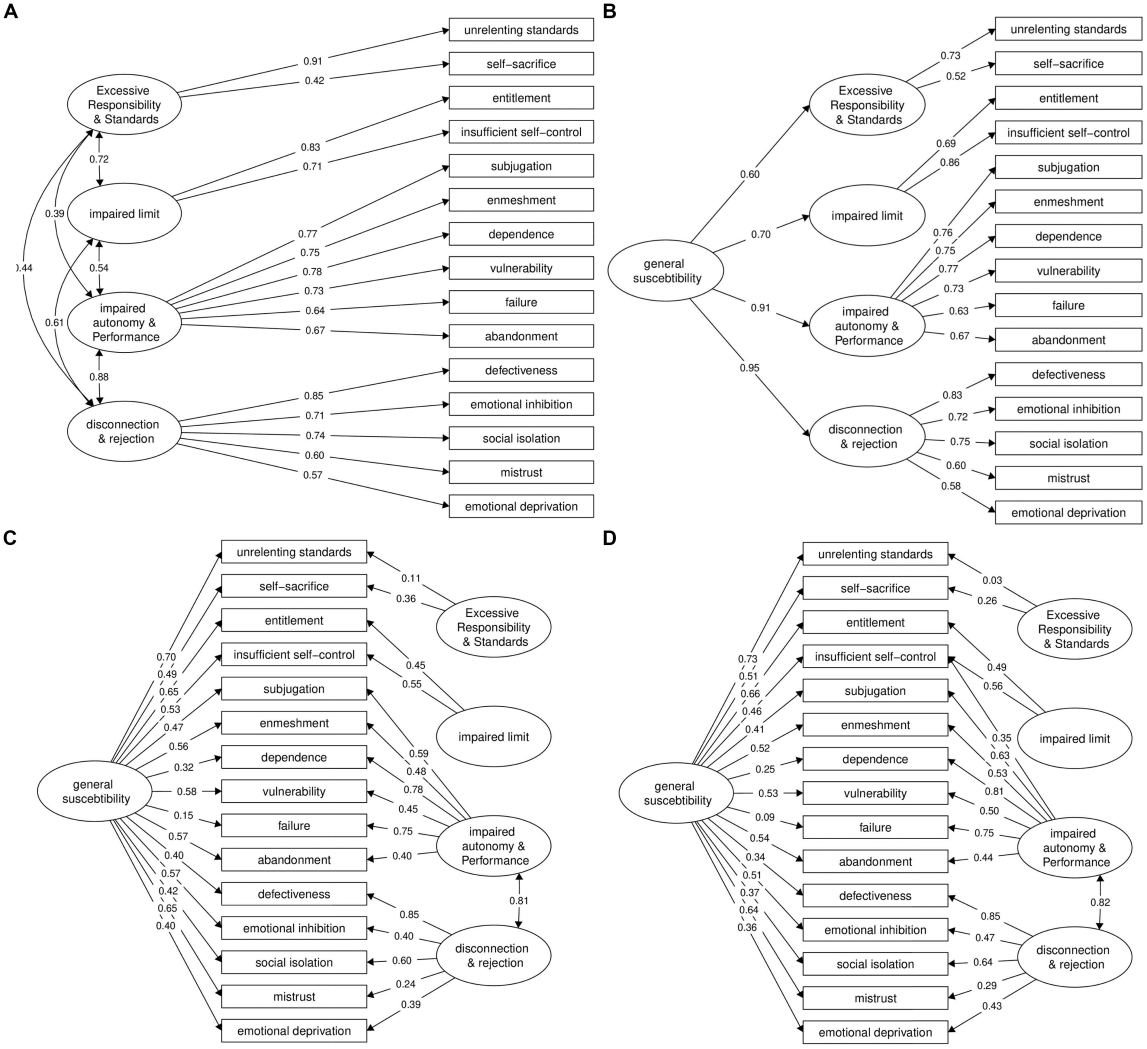
Panel **(A)**: a correlated factor model based on the current schema model; Panel **(B)**: a hierarchical factor model based on the current schema model and a third-order factor; Panel **(C)**: a bifactor model based on the current schema model; Panel **(D)**: a modified version of the Panel **(C)** bifactor model.

The original five-domain model showed a poor fit (Table 1). The intercorrelation among latent factors was also problematically high, with a median of 0.72 and three correlations higher than 0.88. The revised four-domain structure also showed a poor fit (Table 1; Figure 2A). However, the global fit was slightly better and the intercorrelation among domains was less problematic. Using Vuong’s likelihood ratio test for unnested models and information criteria indices, the evidence on the superiority of the four-domain model was weak, *z* = 0.86, *p* = 0.196; ΔAIC = −26.7, 95% CI [−69.5, 16.2]; ΔBIC = −40.4 [−83.2, 2.5].

We investigated a modification recently suggested, allowing four secondary loadings (Figure 2 in Bach et al., 2018). However, only one of these secondary loadings was significant (Insufficient Self-Control onto Impaired Autonomy, *β* = 40, *p* < 0.001); the fit was better but not satisfactory (Table 1, 4-factor b).

Next, we fitted a bifactor model, with a general factor and the four domains. The solution was improper, with four negative error variance terms. Allowing Disconnection & Rejection to freely covary with Impaired Autonomy resolved the issue (Figure 2C). Compared to the four-domain model, the fit substantially improved; Δχ^2^(10) = 103.3, *p* < 0.001 (see Table 1). The general factor accounted for 27% and the four specific factors altogether accounted for 28% of the total variance across items. Allowing Insufficient Self-Control to cross-load on Impaired Autonomy further improved the fit (Table 1; Figure 2D).

Finally, we fitted a higher-order model with four domains and one higher-order factor loaded by these domains (see Table 1 for fit indices). Compared to the bifactor model, the fit was substantially worse, Δχ^2^(11) = 124.8, *p* < 0.001, ΔAIC = 113, ΔBIC = 71. The model had other problems too. The loading on Disconnection & Rejection was *β* = 0.95, leading to a nonsignificant variance in Disconnection & Rejection domain; this means that with the common third-order factor in the model, there is little utility for this domain. The loading on Impaired Autonomy was also too high, *β* = 0.91 (Figure 2B). However, the general factor accounted for 64% of the total variance in the four domains.

### 3.3 The relation between schemas and facial emotion recognition

The overall success rate in the emotion recognition task across all conditions was 88%; it was 94% for fear, 80% for both anger and disgust, 88% for both sadness and neutral, and 99% for happiness. The overall rate in masked and non-masked conditions was 86 and 91%, respectively.

#### 3.3.1 The contribution of specific schemas

At the schema level, none of the 15 schemas were significantly correlated with the total score on emotion recognition, nor on the total scores in masked or non-masked conditions (all *p*s > 0.05). We then assessed their correlation separately with each emotion. Among the 90 correlations (15 schema times 6 emotional states), only four were significant (Supplementary Table S2). Given an alpha level of 0.05 and 90 tests, this is approximately the number of significant results expected by chance (90 × 0.05 = 4.5).

#### 3.3.2 The contribution of schema domains

We investigated the relation of schema domains to facial emotion recognition; both were included as latent variables. Although the fit was not good (χ^2^[179] = 467.7, *p* < 0.001; CFI = 0.838, RMSEA = 0.085 [0.079, 0.98], SRMR = 0.075), there was no problematic residual covariance between indicators of schema-domains and emotion recognition; therefore, the model seemed appropriate for our purpose. The correlations between schema domains and emotion recognition were all nonsignificant (all |r| < 0.12, all *p*s > 0.2). We further explored the relation separately for each emotion. Better recognition of anger was significantly correlated with Disconnection & Rejection, *r* = 0.16, *p* = 0.014, and Impaired Autonomy, *r* = 0.14, *p* = 0.034. When the general susceptibility factor was included in a bifactor model, the correlation of both domains reduced and became nonsignificant, *p* > 0.2.

#### 3.3.3 The contribution of the general susceptibility factor

Finally, the general susceptibility factor was significantly related to detecting anger, *r* = 0.14, *p* = 0.013. Its relation to detecting other emotions, the total score on emotion recognition, or the recognition of negative emotions (i.e., fear, anger, disgust, and sadness) was not significant, all *p*s > 0.1.

## 4 Discussion

This study had two objectives. First, to assess the structure of schemas, mainly focusing on the schema domains and the common susceptibility factor. Second, it investigates how schemas are related to facial emotion recognition.

### 4.1 The structure of schemas: the presence of a general factor

Regarding our first objective, while the 15-factor first-order structure was confirmed, the original and the revised models of the second-order structure showed a problematic fit. The original five-domain model seemed more problematic; it had a poor fit along with very high intercorrelations among domains. Such high intercorrelations raise doubt whether the domains indeed represent distinct factors, and even so, it would be of little use in applied research, as there is very small domain-specific variance to work with.

The revised four-factor model showed a better yet unsatisfactory fit. The unsatisfactory fit of the four-domain model is also shown in a meta-analytic CFA investigation in which the model was fitted to a pooled covariance matrix meta-analytically drawn from 27 samples (Thimm, 2022). Furthermore, in a recent study with a large sample (*N* ≈ 2,300), the four-, five-, and single-domain models all showed similarly poor fit according to CFI and χ^2^/df and similarly good fit according to SRMR and RMSEA. However, another study on Iranian psychiatric patients reported an acceptable fit for the original five-domain model and a nearly acceptable fit for the four-domain model (Khosravani et al., 2020).

This study, along with previous evidence, suggests that a single susceptibility factor is consistently extractable from YSQ. At the item level, a single factor accounted for 28% of the total variance; at the schema level, a single factor accounted for 40% of the total variance; and at the domain level, a single factor accounted for 64% of the variance in the four domains (90% of the variance in Disconnection & Rejection). This means that any relation of domains to other constructs can be due to the relation of the general factor to those constructs. The same issue is present in relation between schemas and other outcomes but with a lower magnitude.

The nature of the common susceptibility factor requires further investigation. The shared variance likely reflects the influence of neuroticism. Previous evidence suggests that all EMSs are correlated with neuroticism, except for Entitlement and Self-Sacrifice. Interestingly and in concordance with these findings, in this study, the same two schemas had the lowest loading of the general factor; while the total item variance explained by the general factor was 28% across all items, the total variance explained by the general factor for Entitlement was 11% and for Self-Sacrifice was 9%. The common method variance (Podsakoff et al., 2003) is another possible explanation for the empirical presence of the general factor. Also, a combination of these two is possible.

There is another important issue to consider. If we accept that the bifactor model is the correct one in the population, then YSQ may need further revision to enhance the reliability of schemas when the variance due to the common factor is partialled out. In this and a previous study (Kriston et al., 2012), with the inclusion of a general factor, the reliability of specific factors was substantially reduced, and for some schemas (in this study for most of them), it fell below the conventional threshold. The low reliability makes it difficult to investigate the contribution of a specific schema to other constructs of interest. One potential way for such a revision is to use the original long-form schema questionnaire to perform an item that maximizes the schema-specific variance in the context of the bifactor model. Notably, such a revision may also help with finding the higher-order structure that is of incremental validity and practical utility.

However, ignoring the shared variance among schemas, and exploring the relation between schemas and other constructs can be misleading. A consistently found relation between a schema and an outcome may be indeed due to the influence of general susceptibility (neuroticism perhaps). Also, given that the majority of the variance in domains (especially Disconnection & Rejection and Impaired Autonomy) can be attributed to the general factor, any association between domains and other constructs must be interpreted with great caution.

Before ending our discussion regarding the second-order structure, it is worth noting that we can think of such a classification in a different way. We think of it as a merely practical tool. Regardless of their correspondence with real entities, the domains may provide a structure to the schemas so that the therapists do not get lost between the large number of schemas. Notably, Lockwood and Perris (2012, p. 58) cautioned against firm conclusions about these higher-order factors being real entities.

### 4.2 Association of schemas to facial emotion recognition

Regarding our second objective, we had tentative evidence for a positive association between the general factor of schemas and anger recognition. Disconnection & Rejection was also related to anger recognition; however, when the variance of the general factor was partialled out, the correlation reduced to a nonsignificant level. We could not find reliable evidence on the association of other domains or schemas with facial emotion recognition. So far, the evidence from this and the two previous studies do not provide a clear picture.

In the first study on this topic, Csukly et al. (2011) investigated the association between four schema domains (extracted in an exploratory analysis) and seven facial expressions among inpatients with major depression. Three significant negative associations were found: one domain (heavily loaded by Dependence, Failure, and Subjugation) was related to weaker detection of happiness, another (heavily loaded by Entitlement and Insufficient Self-Control) to detecting sadness, and a third (heavily loaded by Self-Sacrifice and Unrelenting Standards) to detecting fear; but the effect sizes were small.

We could not replicate these results. There are several possible explanations. First, their sample were inpatients with severe depression, which can influence the results; according to the same study, depressed individuals scored approximately 1 *SD* lower than the control group on the emotion recognition task. Second, it seems that a total of 28 tests were done, which inflates the type-I error rate. Third, it is possible that the domain factors were assessing different constructs, as the different loading patterns suggest. Furthermore, we used a confirmatory approach which does not allow for cross-loadings while they used an exploratory one which allows. While the specific schemas were similar, different constructions of latent factors can lead to different relations to emotion recognition.

More recently, Panić et al. (2022) conducted a similar study on a student sample. They reported significant and fairly high associations between all the five domains of schemas (from the original structure) and overall recognition of negative emotions. The magnitude was fairly similar for all the five domains (ranging from *β* = −0.32 to *β* = −0.41). Given the high correlation among the five domains and the similarity of their associations with emotion recognition, it is very likely that there is a shared aspect from these domains that is associated with emotion recognition, i.e., the general factor. Notably, in contrast to Csukly et al. (2011), none of the domains were significantly related to the recognition of happiness or sadness. Also, only one domain was correlated with fear; even so, the domains correlated with fear recognition were substantially different across the two studies.

Summing up, current evidence on the association between schemas and facial emotion recognition is mixed. This study, which had a larger sample size, showed mostly null results; the other two, while supporting some significant relations, they support different patterns of associations. The contradiction among pieces of evidence may partly stem from a combination of low power and numerous hypothesis testing. The low power can be partly due to the low reliability in the measurement of emotion recognition tasks. In this study, the reliability of the total scores was acceptable at best (ICC = 0.61), and the reliability for scores on separate emotions was poor (with ICCs ranging from 0.15 to 0.55). The other two studies did not report information on reliability, but they likely had similar or slightly higher reliability coefficients, given the similarity of tasks. Low reliability attenuates the relationship and reduces the power; when combined with multiple hypothesis testing, this can lead to random significant findings that contradict across studies.

Another possible explanation for the null results is the ceiling effect due to the simplicity of the emotion recognition task, which does not match real-life situations. In real-life situations, facial expressions usually last for a few seconds at most (long expressions are likely fake; Ekman and Friesen, 1975, p. 14). Also, in contrast to the tasks conducted in these studies, the observers’ attention is usually divided within real-life situations. This ceiling effect may have affected us more than Csukly et al. (2011), as their overall recognition rate was 48.3%, likely due to their recognition task being more difficult (They used different levels of task intensity). Panić et al. (2022) did not report the recognition rate, so the comparison was not possible.

Finally, and from another perspective, the behavioral response can be different for different individuals with the same schema (Young et al., 2003, p. 32); one with an Abandonment schema may avoid cues of rejection; another may get more vigilance; and a third person may use two different strategies at different time points (p. 33). While these factors are ignored, the relation may not be observable.

### 4.3 Limitations

Several limitations must be considered. Despite our sample size being fair for our main objectives, complex models such as bifactor models may need larger samples for accurate parameter estimation; this may be why some models resulted in improper solutions. Also, the majority of our sample were women (88%); therefore, we must be cautious in generalizing these results to men. Furthermore, the sampling was done through social media, and the majority were young adults (the median age was 22) with some level of university education. In the emotion recognition task, we used static pictures with fairly intense expressions; this usually is not the case in real-life situations. However, these static pictures are the validated tools that were used in previous similar studies. All these aspects can affect the generalizability of our results to other populations and to real-life situation.

Regarding the internal validity, using extreme static emotional expressions makes the task easy for participants, leading to a high success rate and, hence, the ceiling effect, which can mask the real effects by attenuating the relation. Furthermore, the reliability of scores on emotion recognition was not good. Low reliability can lead to lower statistical power. Also, it can lead to the underestimation of the relationships between constructs. Of course, our analytic methods, i.e., structural equation modeling, can partly overcome these shortcomings by accounting for unreliability.

### 4.4 Recommendations and future directions

The literature on the higher-order structure of schemas is in its infancy. Researchers have begun using domain scores that still lack clear meaning, rationale, and validity; especially, as there is evidence that they may represent some sort of general susceptibility. Our discussion on this general factor further points to another word of caution: any bivariate relation of a schema to another construct may be attributed to this general factor. To make sure that this is not the case, this common factor needs to be partialled out, e.g., in a bifactor model. Relevant to this point, the schema questionnaire likely benefits from a revision that enhances the schema-specific variance; such a revision enhances the reliability of schemas when the common factor is partialled out. Furthermore, while we provided notable evidence on the presence of a general factor, its nature is still unclear and requires further exploration. Investigations into the relation of the general factor with other related constructs such as neuroticism may provide a better understanding. Finally, the hypothesis of parallel structure of needs and schemas and their correspondence is interesting, yet further examination is warranted.

Regarding the contribution of schemas to facial emotion recognition, the literature likely benefits from studies with larger samples, better assessment procedures, and more realistic tasks. Using video instead of static pictures may be useful in this regard. Finally, the reliability of emotion recognition assessment warrants further attention.

## 5 Conclusion

While there is an emerging trend to use domain scores from Yaung’s schema questionnaire, this study puts its validity into question. Substantial evidence from this and previous studies indicate that all schemas have something in common, what we here call the *general susceptibility factor*. We showed that domain scores mostly represent this general factor rather than unique aspects of clusters of schemas. This general factor also has a substantial share from each schema, therefore any bivariate correlation between schemas and other variables may be in fact due to this shared aspect and not the specific schema. We suggest a bifactor model in which the general and the specific factors are extracted simultaneously. Then, the contribution of the general factor and specific schemas can be evaluated properly. However, we further showed that the schema questionnaire likely benefits from a revision, as the schemas show poor reliability in the presence of the general factor.

We used this bifactor model in our exploration of the association between schemas and facial emotion recognition. Our results showed that when the general susceptibility factor was not included in our measurement model, two domains showed significant correlations with recognizing anger, but when the general factor was included, it was the only significant contributor. This provides an instance for our argument that ignoring the general factor may mislead our understanding of schemas’ contributions.

## Data availability statement

The raw data supporting the conclusions of this article will be made available by the authors, without undue reservation.

## Ethics statement

The studies involving humans were approved by the ethics committee at Shahid Beheshti University (IR.SBU.REC.1401.007). The studies were conducted in accordance with the local legislation and institutional requirements. Written informed consent for participation in this study was provided by the participants’ legal guardians/next of kin.

## Author contributions

ST: Conceptualization, Data curation, Investigation, Methodology, Writing – original draft. AZ-T: Conceptualization, Formal analysis, Investigation, Methodology, Writing – original draft. MF: Conceptualization, Investigation, Methodology, Project administration, Supervision, Writing – review & editing. HF: Conceptualization, Formal analysis, Methodology, Writing – review & editing.
