# Supplementary material for: Decomposing the variance in early maladaptive schemas: the major role of one general factor, the minor role of domains, and their differential relations to facial emotion recognition
Source: Front Psychol. 2024 May 15;15:1342480. doi: 10.3389/fpsyg.2024.1342480 (PMC11134781; doi:10.3389/fpsyg.2024.1342480)
Supplement: Supplementary file 1 [file Table_1.DOCX]

**Supplementary 1**

**S1**

*Means, standard deviations, and reliability estimates for schemas and their intercorrelations*

| Variable | 1 | 2 | 3 | 4 | 5 | 6 | 7 | 8 | 9 | 10 | 11 | 12 | 13 | 14 | 15 |
| --- | --- | --- | --- | --- | --- | --- | --- | --- | --- | --- | --- | --- | --- | --- | --- |
| 1. emotional deprivation | – |  |  |  |  |  |  |  |  |  |  |  |  |  |  |
| 2. abandonment | .37^**^ | – |  |  |  |  |  |  |  |  |  |  |  |  |  |
| 3. mistrust | .39^**^ | .51^**^ | – |  |  |  |  |  |  |  |  |  |  |  |  |
| 4. social isolation | .44^**^ | .37^**^ | .48^**^ | – |  |  |  |  |  |  |  |  |  |  |  |
| 5. defectiveness | .50^**^ | .55^**^ | .48^**^ | .66^**^ | – |  |  |  |  |  |  |  |  |  |  |
| 6. failure | .31^**^ | .41^**^ | .22^**^ | .40^**^ | .66^**^ | – |  |  |  |  |  |  |  |  |  |
| 7. dependence | .33^**^ | .55^**^ | .37^**^ | .49^**^ | .72^**^ | .70^**^ | – |  |  |  |  |  |  |  |  |
| 8. vulnerability | .39^**^ | .57^**^ | .52^**^ | .41^**^ | .56^**^ | .45^**^ | .53^**^ | – |  |  |  |  |  |  |  |
| 9. enmeshment | .41^**^ | .54^**^ | .44^**^ | .45^**^ | .60^**^ | .44^**^ | .59^**^ | .68^**^ | – |  |  |  |  |  |  |
| 10. subjugation | .51^**^ | .56^**^ | .51^**^ | .44^**^ | .62^**^ | .53^**^ | .59^**^ | .61^**^ | .64^**^ | – |  |  |  |  |  |
| 11. self-sacrifice | .24^**^ | .37^**^ | .38^**^ | .14^*^ | .21^**^ | .13^*^ | .19^**^ | .37^**^ | .34^**^ | .41^**^ | – |  |  |  |  |
| 12. emotional inhibition | .46^**^ | .40^**^ | .45^**^ | .62^**^ | .52^**^ | .38^**^ | .46^**^ | .52^**^ | .56^**^ | .51^**^ | .24^**^ | – |  |  |  |
| 13. unrelenting standards | .25^**^ | .37^**^ | .46^**^ | .34^**^ | .22^**^ | .11 | .19^**^ | .40^**^ | .39^**^ | .26^**^ | .40^**^ | .45^**^ | – |  |  |
| 14. entitlement | .20^**^ | .39^**^ | .47^**^ | .36^**^ | .32^**^ | .21^**^ | .23^**^ | .33^**^ | .37^**^ | .25^**^ | .27^**^ | .43^**^ | .59^**^ | – |  |
| 15. insufficient self-control | .33^**^ | .42^**^ | .35^**^ | .45^**^ | .44^**^ | .40^**^ | .49^**^ | .45^**^ | .49^**^ | .44^**^ | .22^**^ | .54^**^ | .39^**^ | .60^**^ | – |
| *M* | 9.82 | 12.26 | 11.45 | 9.62 | 7.61 | 8.33 | 7.85 | 9.85 | 9.75 | 10.91 | 17.84 | 11.55 | 17.87 | 15.14 | 13.11 |
| *SD* | 5.28 | 5.65 | 5.29 | 4.84 | 3.58 | 4.3 | 3.6 | 5.19 | 4.96 | 4.6 | 5.5 | 5.9 | 6.18 | 5.65 | 5.9 |
| ω | .87 | .86 | .84 | .85 | .85 | .92 | .87 | .87 | .79 | .79 | .80 | .85 | .81 | .79 | .85 |
| ω – bifactor model^(a)^ | .53 | .42 | .49 | .41 | .15 | .5 | .26 | .32 | .23 | .18 | .71 | .32 | .67 | .6 | .51 |

*Note*. *N* = 233. ω = McDonald’ omega for internal consistency reliability; AVE = average variance extracted.

(a) In the bifactor mode, the ω coefficient for the general factor was .83.

^*^ *p* < .05. ^**^ *p* < .01.

**S2**

*Bivariate correlation among schemas and facial emotion recognition*

| Maladaptive Schema |  | Masking | | |  | Emotion type | | | | | |
| --- | --- | --- | --- | --- | --- | --- | --- | --- | --- | --- | --- |
|  |  | Overall | Masked face | Non-masked |  | Fearful | Neutral | Disgust | Angry | Happy | Sad |
| Emotional deprivation |  | .02 | -.01 | .06 |  | .04 | -.09 | .01 | .11 | -.08 | .02 |
| Abandonment |  | .08 | .12 | .00 |  | -.02 | .07 | .06 | .07 | .06 | .02 |
| Mistrust |  | .06 | .02 | .10 |  | .04 | -.02 | -.01 | .10 | .07 | .05 |
| Social isolation |  | .04 | -.03 | .13 |  | -.07 | -.04 | .01 | .13^*^ | -.02 | .05 |
| Defectiveness |  | .09 | .05 | .12 |  | .01 | -.01 | .07 | .15^*^ | .01 | .03 |
| Failure |  | -.01 | -.04 | .04 |  | -.01 | -.13^*^ | -.01 | .11 | -.01 | .00 |
| Dependence |  | .06 | .05 | .05 |  | .01 | -.05 | .04 | .12 | .01 | .03 |
| Vulnerability |  | .07 | .04 | .07 |  | .01 | -.04 | .05 | .11 | .05 | .04 |
| Enmeshment |  | .06 | .02 | .08 |  | -.03 | -.02 | .02 | .11 | .00 | .05 |
| Subjugation |  | .00 | -.03 | .03 |  | -.03 | -.11 | .03 | .09 | .02 | -.04 |
| Self-sacrifice |  | -.04 | .02 | -.10 |  | .06 | -.14^*^ | .09 | -.10 | .00 | -.01 |
| Emotional inhibition |  | -.07 | -.09 | -.02 |  | -.10 | -.09 | .01 | .06 | -.09 | -.10 |
| Unrelenting standards |  | .07 | .06 | .06 |  | .02 | .00 | .10 | .06 | .02 | .02 |
| Entitlement |  | -.06 | -.07 | -.02 |  | -.04 | -.04 | -.12 | .09 | -.06 | -.05 |
| Insufficient self-control |  | -.06 | -.07 | -.03 |  | .03 | -.16^*^ | -.02 | .11 | -.09 | -.11 |

*Note*. *N* = 233.

* *p* < .05.

**S3**

*Model implied bivariate relation of schema domains and schema general factor to facial emotion recognition*

| Emotion | Disconnection & Rejection | Impaired Autonomy | Impaired Limit | Extreme Effort | General factor |
| --- | --- | --- | --- | --- | --- |
| Fear | -.04 | -.05 | -.03 | .05 | -.04 |
| Neutral | -.04 | -.08 | -.09 | .02 | -.08 |
| Disgust | .06 | .05 | -.08 | .13 | .05 |
| Anger | .16^*^ | .14^*^ | .12 | .03 | .14^*^ |
| Happiness | -.03 | .02 | -.09 | .04 | -.01 |
| Sadness | .06 | .05 | -.11 | .00 | .03 |
| Total score | .01 | .01 | −.12 | .08 | 0 |

*Note.* *N* = 226. These model-implied correlations are based on different first-order factor models, not the bifactor or higher-order models.

* *p* < .05.
