# Supplementary material for: Decomposing the variance in early maladaptive schemas: the major role of one general factor, the minor role of domains, and their differential relations to facial emotion recognition
Source: Front Psychol. 2024 May 15;15:1342480. doi: 10.3389/fpsyg.2024.1342480 (PMC11134781; doi:10.3389/fpsyg.2024.1342480)
Supplement: Supplementary file 3 [file Table_3.DOCX]

R-script for reproducing the results

**# loading the required packages and the dataset**library(readxl)
library(dplyr)
library(lavaan)
library(semTools)
library(nonnest2)
library(psych)

dat.ful <- read_excel("data.xlsx") # use the file address on your device

**#item-level data cleaning, i.e., detecting random responders**item.mahal <- dat.ful %>%
 select(Y_1_ed:Y_75_ic) %>%
 mahalanobis(center = colMeans(select(dat.ful, Y_1_ed:Y_75_ic)), cov = cov(select(dat.ful, Y_1_ed:Y_75_ic)))

rand.resp <- pchisq(item.mahal, 75, lower.tail = F)

dat <- dat.ful %>%
 filter(rand.resp > .001)

**# CFA: first-order structure**

## 1-factor model
mod.1f <- paste('g =~', paste(colnames(select(dat, Y_1_ed:Y_75_ic)), collapse = ' + '))
fit.1f <- cfa(mod.1f, dat ,estimator = 'MLR')
summary(fit.1f, fit = T, std = T)
nullRMSEA(fit.1f)

## 15-factor model
mod.15f <- '
ed =~ Y_1_ed + Y_2_ed + Y_3_ed + Y_4_ed + Y_5_ed
ab =~ Y_6_ab + Y_7_ab + Y_8_ab + Y_9_ab + Y_10_ab
mt =~ Y_11_mt + Y_12_mt + Y_13_mt + Y_14_mt + Y_15_mt
si =~ Y_16_si + Y_17_si + Y_18_si + Y_19_si + Y_20_si
def =~ Y_21_def + Y_22_def + Y_23_def + Y_24_def + Y_25_def
fal =~ Y_26_fal + Y_27_fal + Y_28_fal + Y_29_fal + Y_30_fal
dep =~ Y_31_dep + Y_32_dep + Y_33_dep + Y_34_dep + Y_35_dep
vul =~ Y_36_vul + Y_37_vul + Y_38_vul + Y_39_vul + Y_40_vul
enm =~ Y_41_enm + Y_42_enm + Y_43_enm + Y_44_enm + Y_45_enm
sub =~ Y_46_sub + Y_47_sub + Y_48_sub + Y_49_sub + Y_50_sub
ss =~ Y_51_ss + Y_52_ss + Y_53_ss + Y_54_ss + Y_55_ss
inh =~ Y_56_inh + Y_57_inh + Y_58_inh + Y_59_inh + Y_60_inh
us =~ Y_61_us + Y_62_us + Y_63_us + Y_64_us + Y_65_us
ent =~ Y_66_ent + Y_67_ent + Y_68_ent + Y_69_ent + Y_70_ent
ic =~ Y_71_ic + Y_72_ic + Y_73_ic + Y_74_ic + Y_75_ic'
fit.15f <- cfa(mod.15f, dat ,estimator = 'MLR')
summary(fit.15f, fit = T, std = T)

## 15-factor model - uncorrelated
fit.15f.orth <- cfa(mod.15f, dat ,estimator = 'MLR', orthogonal = T)
summary(fit.15f.orth, fit = T, std = T)

## bifactor model
mod.bi <- paste(mod.15f, mod.1f, sep = '\n')
fit.bi <- cfa(mod.bi, dat ,estimator = 'MLR', orthogonal = T, std.lv = T)
summary(fit.bi, fit = T, std = T)

## comparing the 15-factor and bifactor models
anova(fit.15f, fit.bi)

**# CFA: second-order structure (schema domains)**## screening data for outliers
schema.mahal <- dat %>%
 select(emo_dep:insuf_self_ctrl) %>%
 mahalanobis(center = colMeans(select(dat, emo_dep:insuf_self_ctrl)), cov = cov(select(dat, emo_dep:insuf_self_ctrl)))
schema.outlier <- pchisq(schema.mahal, 15, lower.tail = F)
dat.schema <- dat %>%
 filter(schema.outlier > .001)
## 1 general second-order factor
mod.1d <-
'g =~ emo_dep + aband + mistrust + so_isolate + defectiveness + failure + dependence + vulner + enmeshment + subjug + self_sacrif + emo_inhib + unrelent_std + entitlement + insuf_self_ctrl

'
fit.1d <- cfa(mod.1d, dat.schema, estimator = 'MLR')
summary(fit.1d, fit = T, std = T)
nullRMSEA(fit.1d)
## 5-domain model
mod.5d <- '
disconnect =~ emo_dep + aband + mistrust + so_isolate + defectiveness
impaired.autonomy =~ failure + dependence + vulner + enmeshment
other.directed =~ subjug + self_sacrif
over.vigilance =~ emo_inhib + unrelent_std
impaired.limit =~ entitlement + insuf_self_ctrl'
fit.5d <- cfa(mod.5d, dat.schema, estimator = 'MLR')
summary(fit.5d, fit = T, std = T)

## 4-domain model
mod.4d <- '
disconnect =~ emo_dep + mistrust + so_isolate + defectiveness + emo_inhib
impaired.autonomy =~ aband + failure + dependence + vulner + enmeshment + subjug
extreme.effort =~ self_sacrif + unrelent_std
impaired.limit =~ entitlement + insuf_self_ctrl'
fit.4d <- cfa(mod.4d, dat.schema, estimator = 'MLR')
summary(fit.4d, fit = T, std = T)

## comparing 5-domain and 4-domain model (using ML estimation and Vuong method for non-nested model comparrison)
fit.4d.ml <- cfa(mod.4d, dat.schema, estimator = 'ML')
fit.5d.ml <- cfa(mod.5d, dat.schema, estimator = 'ML')
vuongtest(fit.4d.ml, fit.5d.ml)
icci(fit.4d.ml, fit.5d.ml)

## 5-domain: full hierarchy (item-level data)
mod.15f.5d <- paste(mod.15f, '
disconnect =~ ed + ab + mt + si + def
impaired.autonomy =~ fal + dep + vul + enm
other.directed =~ sub + ss
over.vigilance =~ inh + us
impaired.limit =~ ent + ic', sep = '\n')
fit.15f.5d <- cfa(mod.15f.5d, dat.schema, estimator = 'MLR')
summary(fit.15f.5d, fit = T, std = T)

## 4-domain: full hierarchy (item-level data)
mod.15f.4d <- paste(mod.15f, '
disconnect =~ ed + mt + si + def + inh
impaired.autonomy =~ ab + fal + dep + vul + enm + sub
extreme.effort =~ ss + us
impaired.limit =~ ent + ic
', sep = '\n')
fit.15f.4d <- cfa(mod.15f.4d, dat.schema, estimator = 'MLR')
summary(fit.15f.4d, fit = T, std = T)

##compare full hierarchy models
fit.15f.5d.ml <- cfa(mod.15f.5d, dat.schema, estimator = 'ML')
fit.15f.4d.ml <- cfa(mod.15f.4d, dat.schema, estimator = 'ML')
vuongtest(fit.15f.5d.ml, fit.15f.4d.ml)
icci(fit.15f.5d.ml, fit.15f.4d.ml)

## examining suggested cross-loadings
mod.4d.cross <- '
disconnect =~ emo_dep + mistrust + so_isolate + defectiveness + emo_inhib + vulner
impaired.autonomy =~ aband + failure + dependence + vulner + enmeshment + subjug + insuf_self_ctrl
extreme.effort =~ self_sacrif + unrelent_std + enmeshment + subjug
impaired.limit =~ entitlement + insuf_self_ctrl'
fit.4d.cross <- cfa(mod.4d.cross, dat.schema, estimator = 'MLR')
summary(fit.4d.cross, std = T, fit = T)

## a modifoed 4-domain model
mod.4d.b <- '
disconnect =~ emo_dep + mistrust + so_isolate + defectiveness + emo_inhib
impaired.autonomy =~ aband + failure + dependence + vulner + enmeshment + subjug + insuf_self_ctrl
extreme.effort =~ self_sacrif + unrelent_std
impaired.limit =~ entitlement + insuf_self_ctrl
entitlement ~~ a*entitlement
a > 0
'
fit.4d.b <- cfa(mod.4d.b, dat.schema, estimator = 'MLR')
summary(fit.4d.b, fit = T, std = T)
my.fit2(fit.4d.b)

## second-order 4-domain bifactor - a
mod.4d.bi <- '
disconnect =~ emo_dep + mistrust + so_isolate + emo_inhib + defectiveness
impaired.autonomy =~ aband + failure + vulner + dependence + enmeshment + subjug
extreme.effort =~ self_sacrif + unrelent_std
impaired.limit =~ entitlement + insuf_self_ctrl
g =~ emo_dep + mistrust + so_isolate + emo_inhib + defectiveness + aband + failure + dependence + vulner + enmeshment + subjug + self_sacrif + unrelent_std + entitlement + insuf_self_ctrl
disconnect ~~ impaired.autonomy
'
fit.4d.bi <- cfa(mod.4d.bi, dat.schema, estimator = 'MLR', orthogonal = T, std.lv = T)
summary(fit.4d.bi, fit = T, std = T)

Plot <- semPlot::semPaths(fit.4d.bi, what = 'std', bifactor = 'g', layout = 'tree3', style = "lisrel", sizeMan = 10, sizeLat = 12,sizeLat2 = 5, residuals = F, nCharNodes = 10, edge.color = "black", edge.label.cex = 1.2, fixedStyle = 1, fade = F, asize = 2, sizeMan2 = 3, esize = 1, label.cex = .5, label.scale = F, exoCov = T, rotation = 2, )
Plot$graphAttributes$Edges$edgeConnectPoints[1:15,2] <- 0.5 * pi
Plot$graphAttributes$Edges$edgeConnectPoints[16:30,2] <- 1.5 * pi
plot(Plot)

##comparing the bifactor 4-domain and correlated 4-domain models
anova(fit.4d, fit.4d.bi)

## second-order 4-domain bifactor - b
mod.4d.bi.b <- '
extreme.effort =~ self_sacrif + unrelent_std
impaired.limit =~ entitlement + insuf_self_ctrl
impaired.autonomy =~ aband + failure + dependence + vulner + enmeshment + subjug + insuf_self_ctrl
disconnect =~ emo_dep + mistrust + so_isolate + emo_inhib + defectiveness
g =~ emo_dep + mistrust + so_isolate + emo_inhib + defectiveness + aband + failure + dependence + vulner + enmeshment + subjug + insuf_self_ctrl + self_sacrif + unrelent_std + entitlement
disconnect ~~ impaired.autonomy
'
fit.4d.bi.b <- cfa(mod.4d.bi.b, dat.schema, estimator = 'MLR', orthogonal = T, std.lv = T)
summary(fit.4d.bi.b, fit = T, std = T)

## a third-order factor
mod.4d.3lvl <- '
disconnect =~ emo_dep + mistrust + so_isolate + defectiveness + emo_inhib
impaired.autonomy =~ aband + failure + dependence + vulner + enmeshment + subjug
extreme.effort =~ self_sacrif + unrelent_std
impaired.limit =~ entitlement + insuf_self_ctrl
G =~ disconnect + impaired.autonomy + extreme.effort + impaired.limit'
fit.4d.3lvl <- cfa(mod.4d.3lvl, dat.schema, estimator = 'MLR')
anova(fit.4d.3lvl, fit.4d.bi)
summary(fit.4d.3lvl, fit = T, std = T)

**# schema and facial emotion recognition**## checking the mearement model of emotion recognition
mod.emo <- '
emotion.rec =~ fear + neut + disgust + anger + happy + sad
'
fit.emo <- cfa(mod.emo, dat.schema, estimator = 'MLR')
summary(fit.emo, fit = T, std = T)

## investigating the bivariate relation between schema domains and overall emotion recognition
mod.domain.emo <- '
disconnect =~ emo_dep + mistrust + so_isolate + defectiveness + emo_inhib
impaired.limit =~ entitlement + insuf_self_ctrl
impaired.autonomy =~ aband + failure + dependence + vulner + enmeshment + subjug
extreme.effort =~ self_sacrif + unrelent_std
emotion.rec =~ fear + neut + disgust + anger + happy + sad
'
fit.domain.emo <- cfa(mod.domain.emo, dat.schema, estimator = 'MLR')
summary(fit.domain.emo, fit = T, std = T)

## investigating the relations between schema domains and overall emotion recognition in a multiple regression model
mod.str <- '
disconnect =~ emo_dep + mistrust + so_isolate + defectiveness + emo_inhib
impaired.limit =~ entitlement + insuf_self_ctrl
impaired.autonomy =~ aband + failure + dependence + vulner + enmeshment + subjug
extreme.effort =~ self_sacrif + unrelent_std
emotion.rec =~ fear + neut + disgust + anger + happy + sad
emotion.rec ~ disconnect + impaired.limit + impaired.autonomy + extreme.effort
'

fit.str <- cfa(mod.str, dat.schema, estimator = 'MLR')
summary(fit.str, fit = T, std = T)
lavInspect(fit.str, 'r2')

## investigating the bivariate relation between schema domains and detecting different emotions
mod.emo.sep <- '
disconnect =~ emo_dep + mistrust + so_isolate + defectiveness + emo_inhib
impaired.autonomy =~ aband + failure + dependence + vulner + enmeshment + subjug
impaired.limit =~ entitlement + insuf_self_ctrl
extreme.effort =~ self_sacrif + unrelent_std
lfear =~ fear
lneut =~ neut
ldisgust =~ disgust
langer =~ anger
lhappy =~ happy
lsad =~ sad
'
fit.emo.sep <- cfa(mod.emo.sep, dat.schema, estimator = 'MLR')
summary(fit.emo.sep, fit = T, std = T)
lavInspect(fit.emo.sep, 'cor.lv')

## the relation of general factor of schemas and detecting different emotions
mod.g.emo <- '
disconnect =~ 1*emo_dep + mistrust + so_isolate + emo_inhib + defectiveness
impaired.autonomy =~ 1*aband + failure + dependence + vulner + enmeshment + subjug
disconnect ~~ 0*impaired.autonomy
g ~~ 0*disconnect + 0*impaired.autonomy
g =~ 1*emo_dep + mistrust + so_isolate + emo_inhib + defectiveness + aband + failure + dependence + vulner + enmeshment + subjug + 1*self_sacrif + unrelent_std + entitlement + insuf_self_ctrl
f =~ fear
n =~ neut
d =~ disgust
a =~ anger
h =~ happy
s =~ sad
'
fit.g.emo <- cfa(mod.g.emo, dat.schema, estimator = 'MLR', std.lv = T)
summary(fit.g.emo, fit = T, std = T)

## g to overal emotion recognition
mod.g.emo <- '
disconnect =~ 1*emo_dep + mistrust + so_isolate + emo_inhib + defectiveness
impaired.autonomy =~ 1*aband + failure + dependence + vulner + enmeshment + subjug
g ~~ 0*impaired.autonomy + 0*disconnect
disconnect ~~ 0*impaired.autonomy
g =~ emo_dep + mistrust + so_isolate + emo_inhib + defectiveness + aband + failure + dependence + vulner + enmeshment + subjug + 1*self_sacrif + unrelent_std + entitlement + insuf_self_ctrl
emotion.rec =~ fear + neut + disgust + anger + happy + sad

'
fit.g.emo <- cfa(mod.g.emo, dat.schema, estimator = 'MLR')
summary(fit.g.emo, fit = T, std = T)
